# Supplementary material for: Discovery of Novel Diarylamide N-Containing Heterocyclic Derivatives as New Tubulin Polymerization Inhibitors with Anti-Cancer Activity
Source: Molecules. 2021 Jul 2;26(13):4047. doi: 10.3390/molecules26134047 (PMC8272053; doi:10.3390/molecules26134047)
Supplement: Supplementary file 1 [file molecules-26-04047-s001.zip › molecules-1233703-supplementary.pdf]

Discovery of Novel Diarylamide *N*-Containing Heterocyclic Derivatives as New Tubulin Polymerization Inhibitors with Anti-Cancer Activity

Xu Liu [1,†], Xiao-Jing Pang [1,†], Yuan Liu [1], Wen-Bo Liu [1], Yin-Ru Li [2], Guang-Xi Yu [1], Yan-Bing Zhang [1], Jian Song [1,*] and Sai-Yang Zhang [1,2,*]

[1] Key Laboratory of Advanced Drug Preparation Technologies (Ministry of Education), Institute of Drug Discovery & Development, School of Pharmaceutical Sciences, Zhengzhou University, Zhengzhou 450001, China; lx012496@163.com (X.L.); summer_pxj@163.com (X.-J.P.); liuyuan9720@163.com (Y.L.); 17698567571@163.com (W.-B.L.); ygx19990110@126.com (G.-X.Y.); zhangyb@zzu.edu.cn (Y.-B.Z.)

[2] School of Basic Medical Sciences, Zhengzhou University, Zhengzhou, 450001, China; 17603868158@163.com

* Correspondence: mumuandzz@163.com (J.S.); saiyangz@zzu.edu.cn (S.-Y.Z.)

† These authors contributed equally to this work.

Contents

^{1}H NMR of compound 15a 2
^{13}C NMR of compound 15a 3
HRMS of compound 15a 4
^{1}H NMR of compound 15b 5
^{13}C NMR of compound 15b 6
^{1}H NMR of compound 15c 7
^{13}C NMR of compound 15c 8
HRMS of compound 15c 9
^{1}H NMR of compound 15d 10
^{13}C NMR of compound 15d 11
HRMS of compound 15d 12
^{1}H NMR of compound 15e 13
^{13}C NMR of compound 15e 14
HRMS of compound 15e 15
^{1}H NMR of compound 15f 16
^{13}C NMR of compound 15f 17
HRMS of compound 15f 18
^{1}H NMR of compound 15g 19
^{13}C NMR of compound 15g 20
HRMS of compound 15g 21
^{1}H NMR of compound 15h 22
^{13}C NMR of compound 15h 23
HRMS of compound 15h 24
^{1}H NMR of compound 19a 25

^{13}C NMR of compound 19a 26
HRMS of compound 19a 27
^{1}H NMR of compound 19b 28
^{13}C NMR of compound 19b 29
HRMS of compound 19b 30
^{1}H NMR of compound 19c 31
^{13}C NMR of compound 19c 32
HRMS of compound 19c 33
^{1}H NMR of compound 19d 34
^{13}C NMR of compound 19d 35
HRMS of compound 19d 36
^{1}H NMR of compound 19e 37
^{13}C NMR of compound 19e 38
HRMS of compound 19e 39

^{1}H NMR of compound 15a

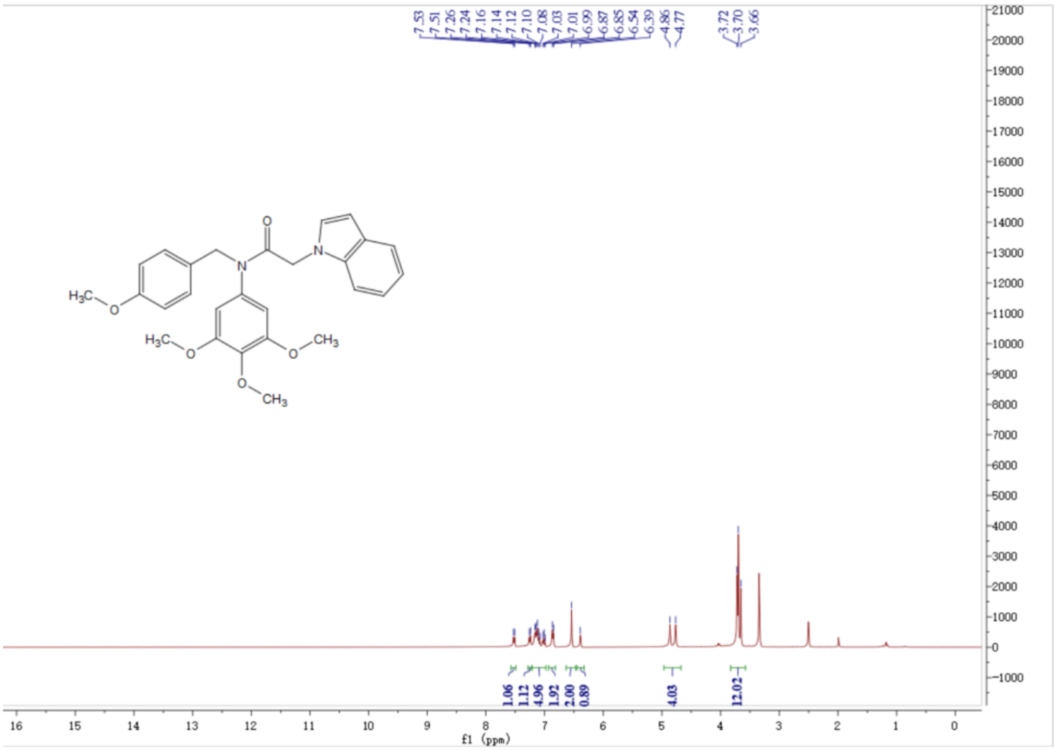

Fig. S1. ^{1}H NMR spectrum of compound **15a** (400 MHz, DMSO-*d6*)

^{13}C NMR of compound 15a

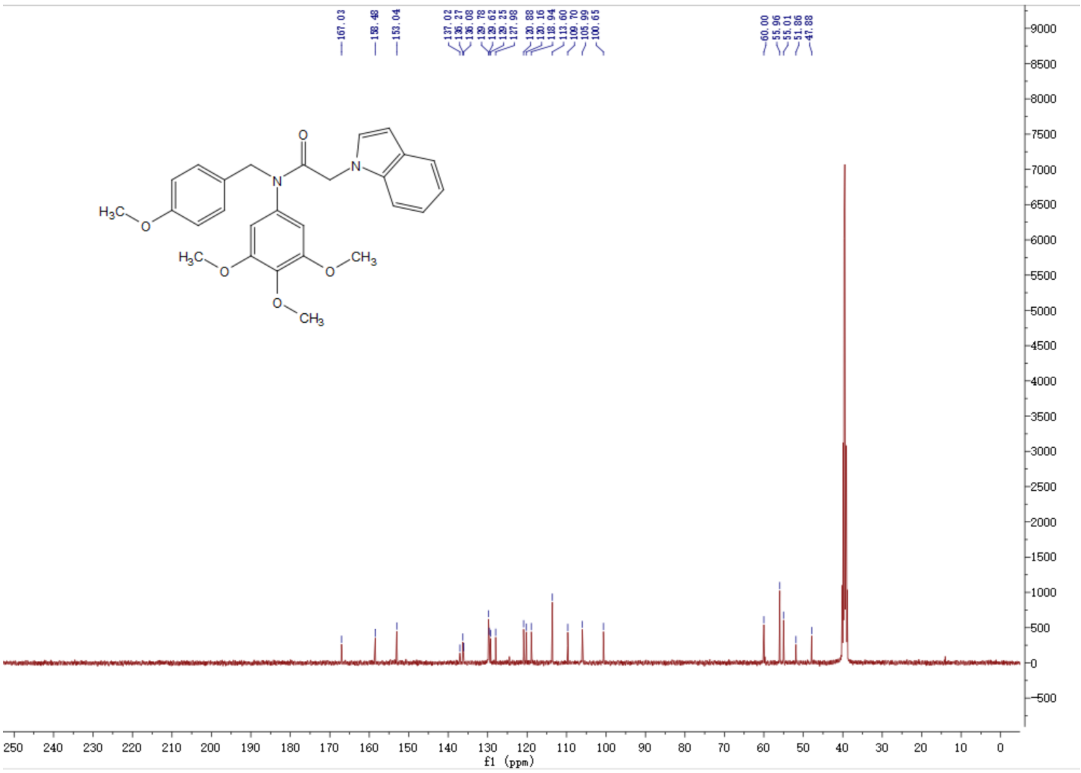

Fig. S2. ^{13}C NMR spectrum of compound **15a** (400 MHz, DMSO-*d6*)

HRMS of compound 15a

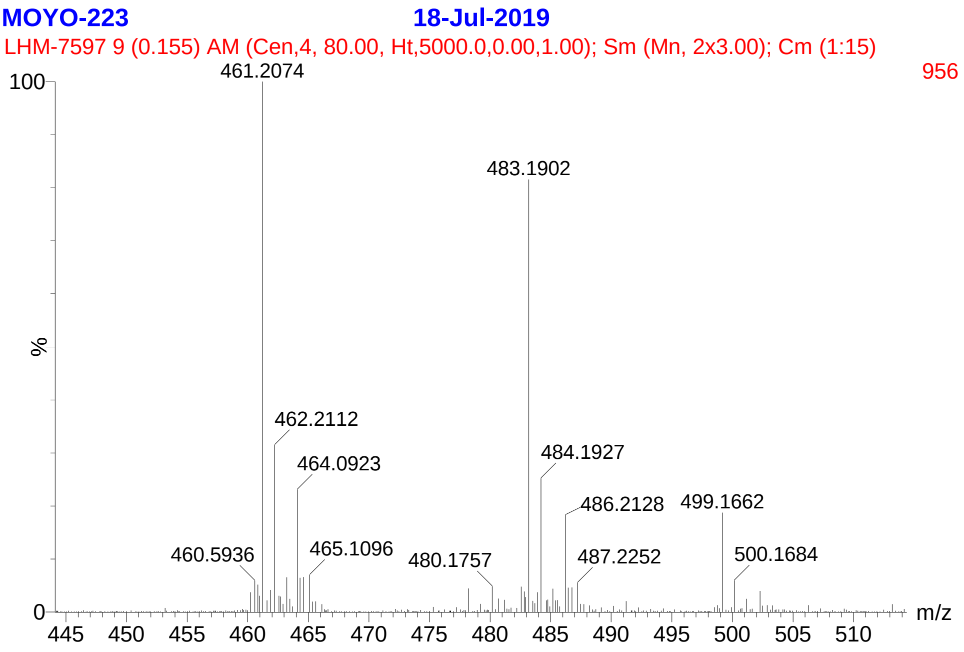

Fig. S3. HRMS spectrum of compound **15a**

^{1}H NMR of compound 15b

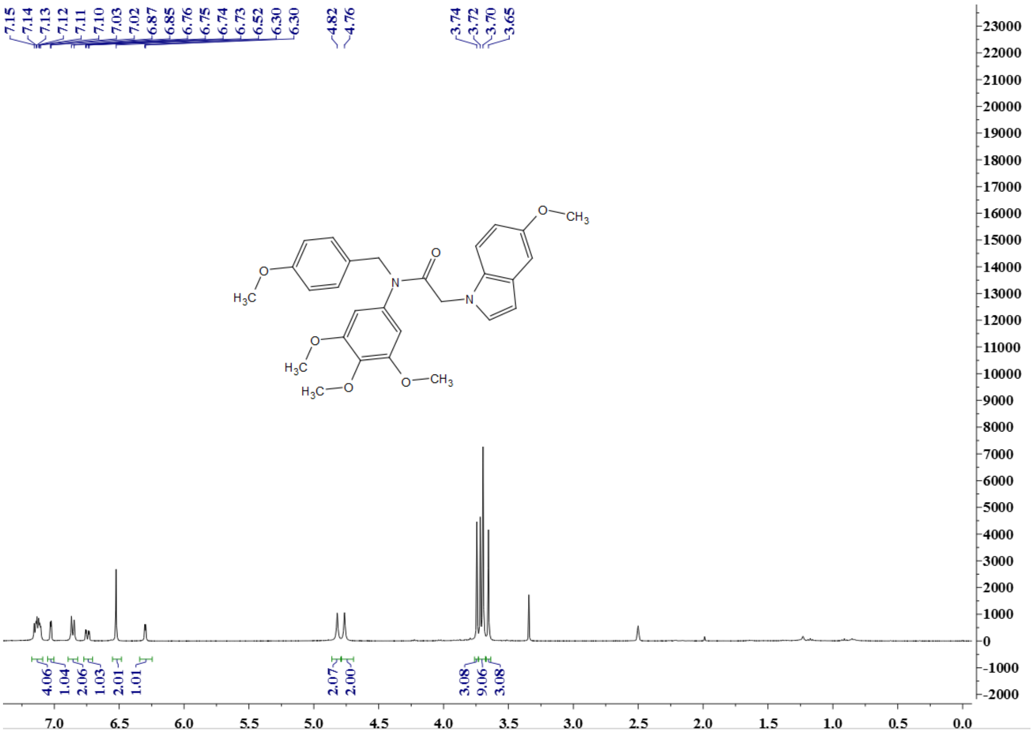

Fig. S4. ^{1}H NMR spectrum of compound **15b** (400 MHz, DMSO-*d6*)

^{13}C NMR of compound 15b

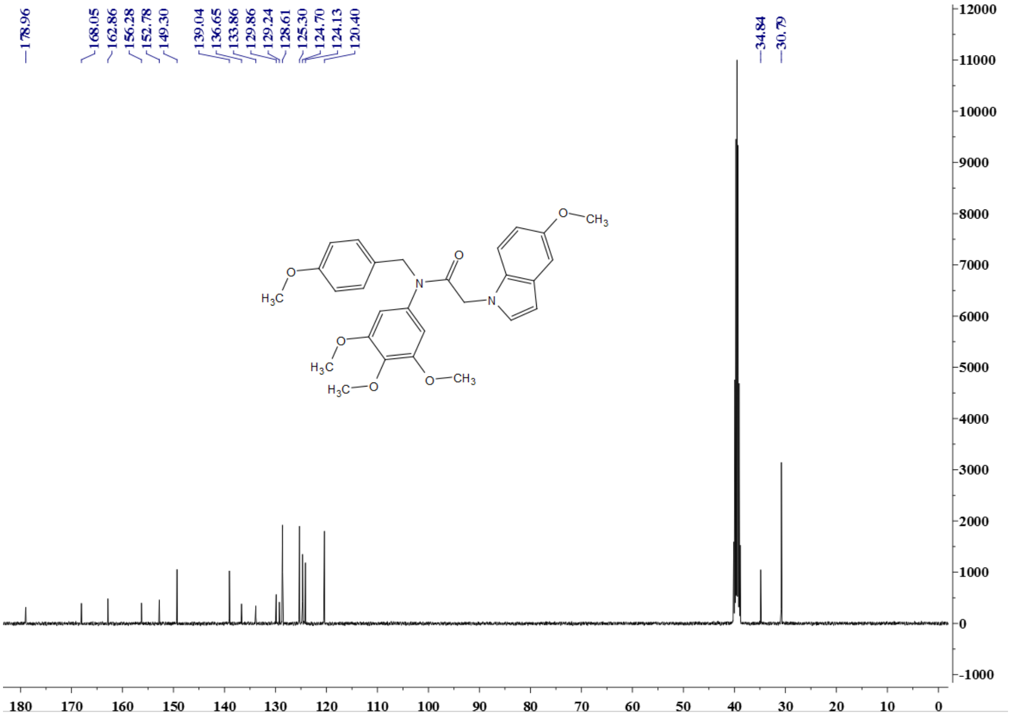

Fig. S5. ^{13}C NMR spectrum of compound **15b** (400 MHz, DMSO-*d6*)

^{1}H NMR of compound 15c

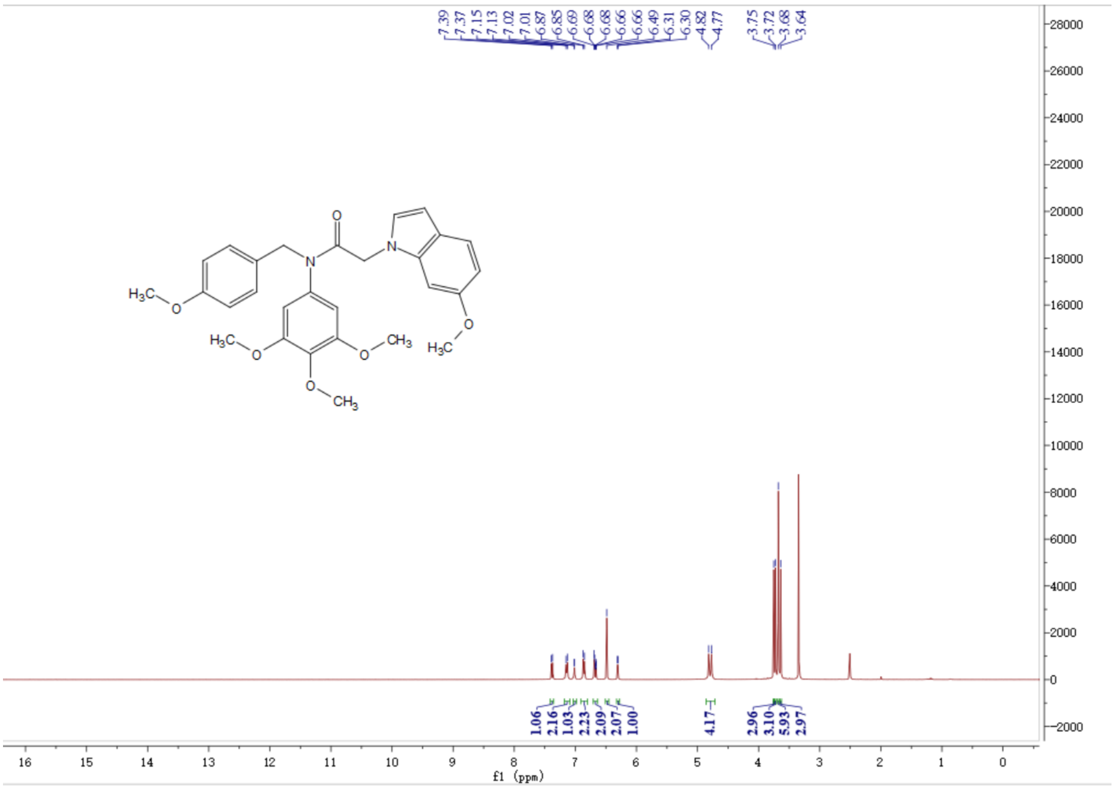

Fig. S6. ^{1}H NMR spectrum of compound **15c** (400 MHz, DMSO-*d6*)

^{13}C NMR of compound 15c

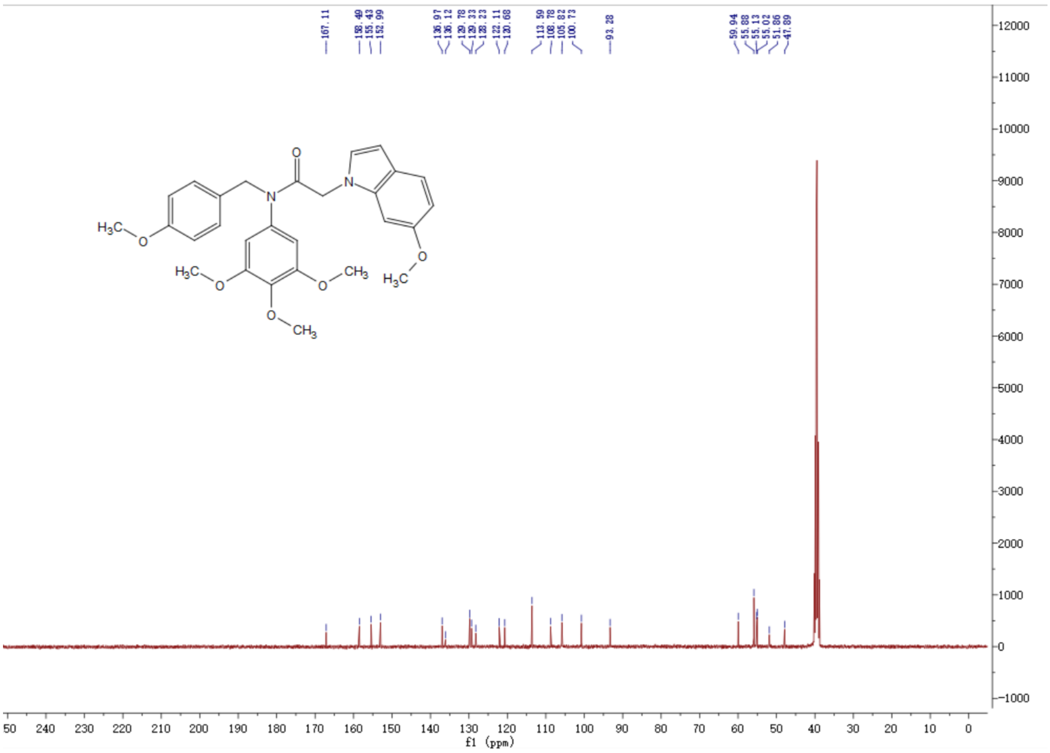

Fig. S7. ^{13}C NMR spectrum of compound **15c** (400 MHz, DMSO-*d6*)

HRMS of compound 15c

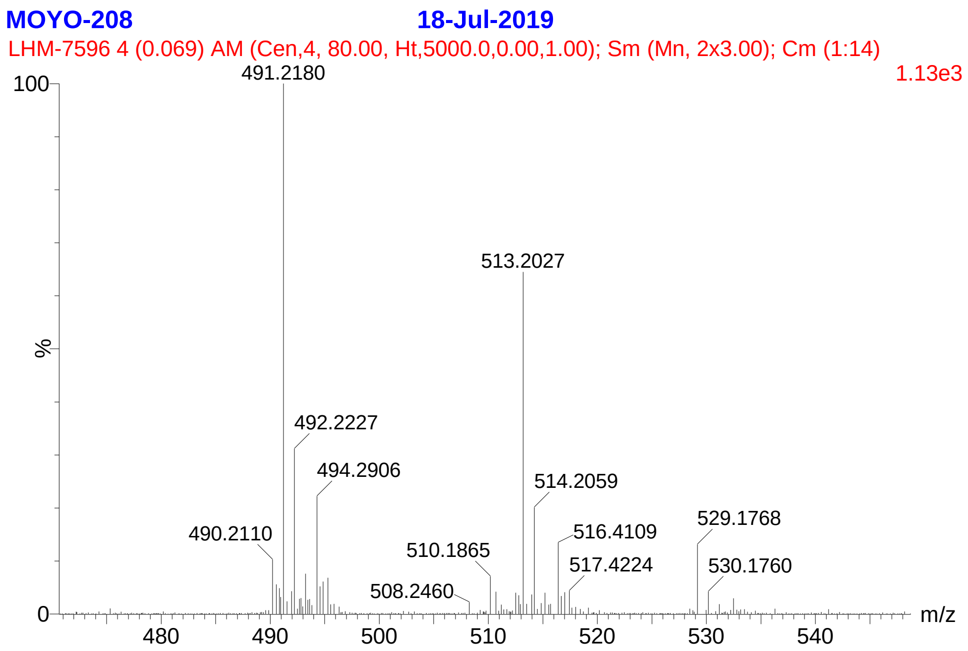

Fig. S8. HRMS spectrum of compound **15c**

^{1}H NMR of compound 15d

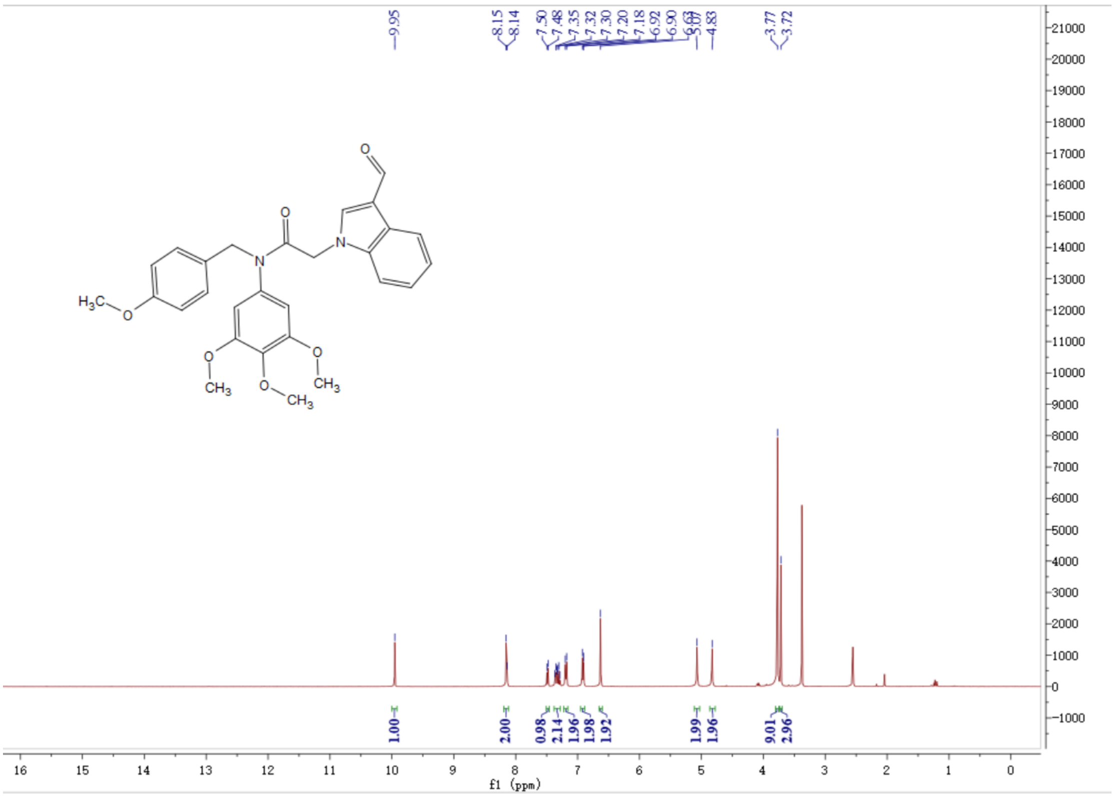

Fig. S9. ^{1}H NMR spectrum of compound **15d** (400 MHz, DMSO-*d6*)

^{13}C NMR of compound 15d

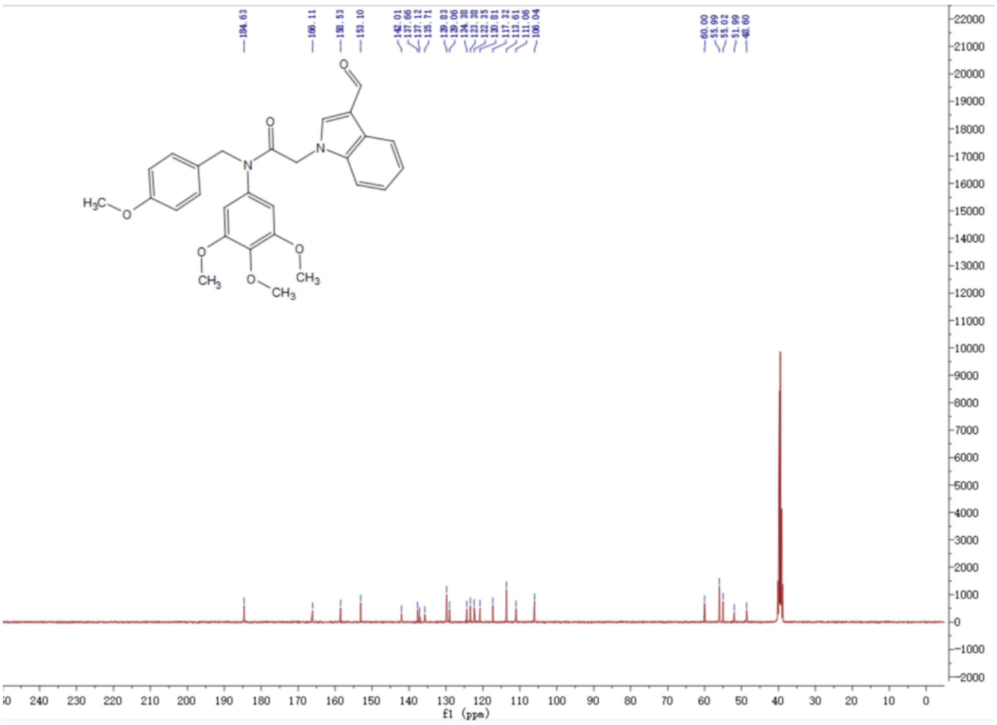

Fig. S10. ^{13}C NMR spectrum of compound **15d** (400 MHz, DMSO-*d6*)

HRMS of compound 15d

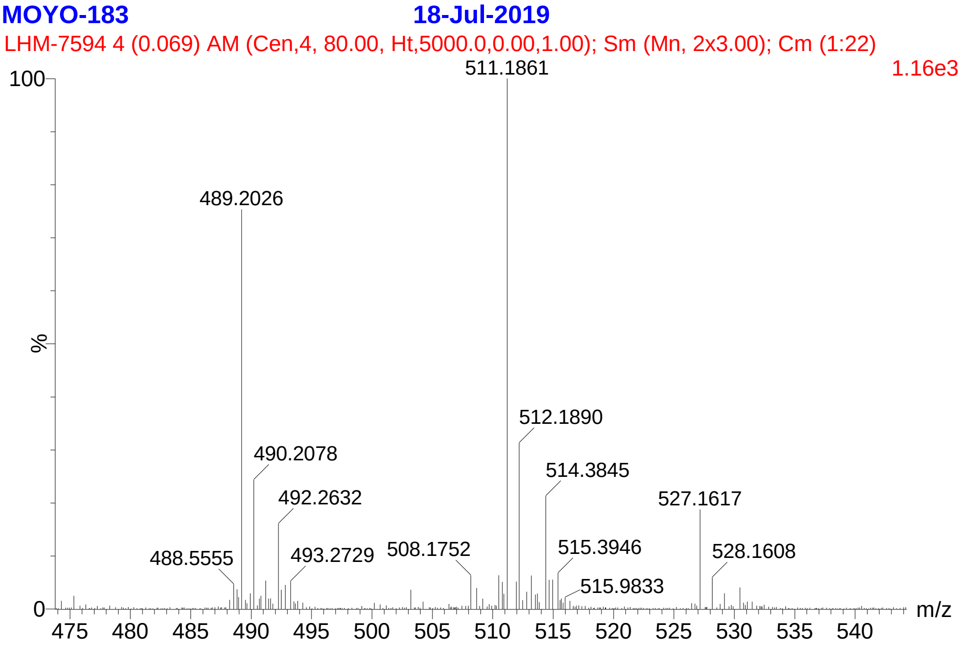

Fig. S11. HRMS spectrum of compound **15d**

^{1}H NMR of compound 15e

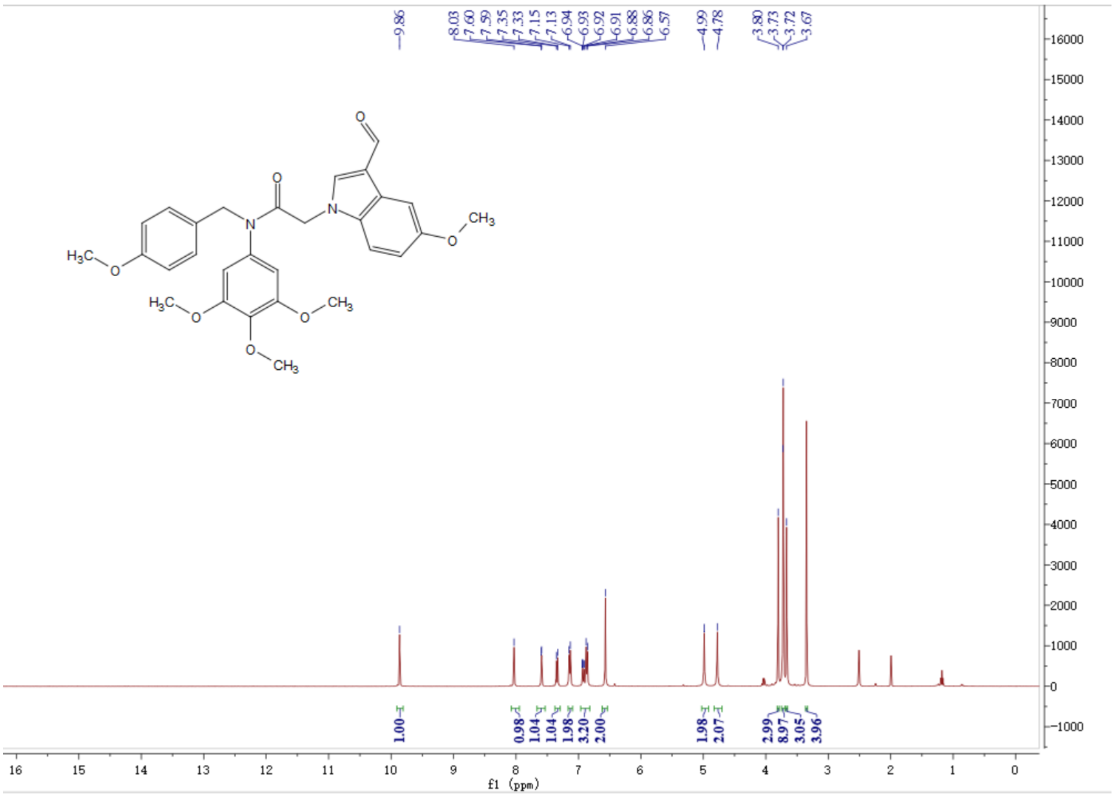

Fig. S12. ^{1}H NMR spectrum of compound **15e** (400 MHz, DMSO-*d6*)

^{13}C NMR of compound 15e

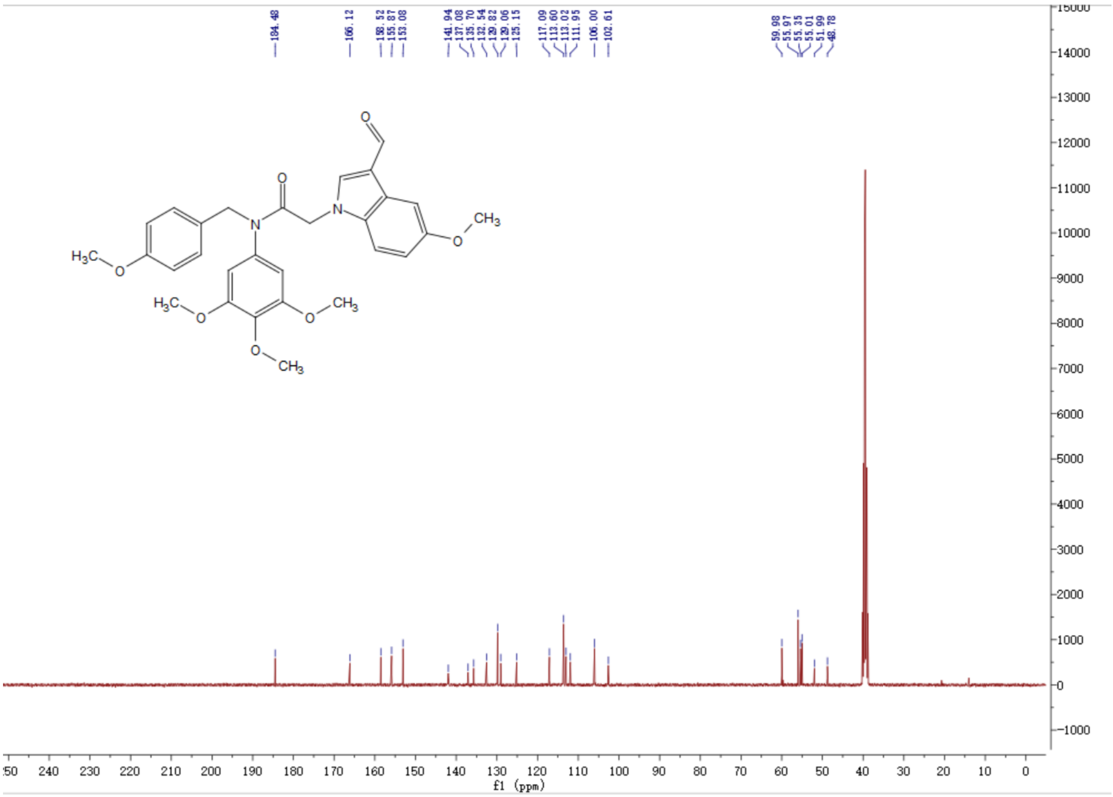

Fig. S13. ^{13}C NMR spectrum of compound **15e** (400 MHz, DMSO-*d6*)

HRMS of compound 15e

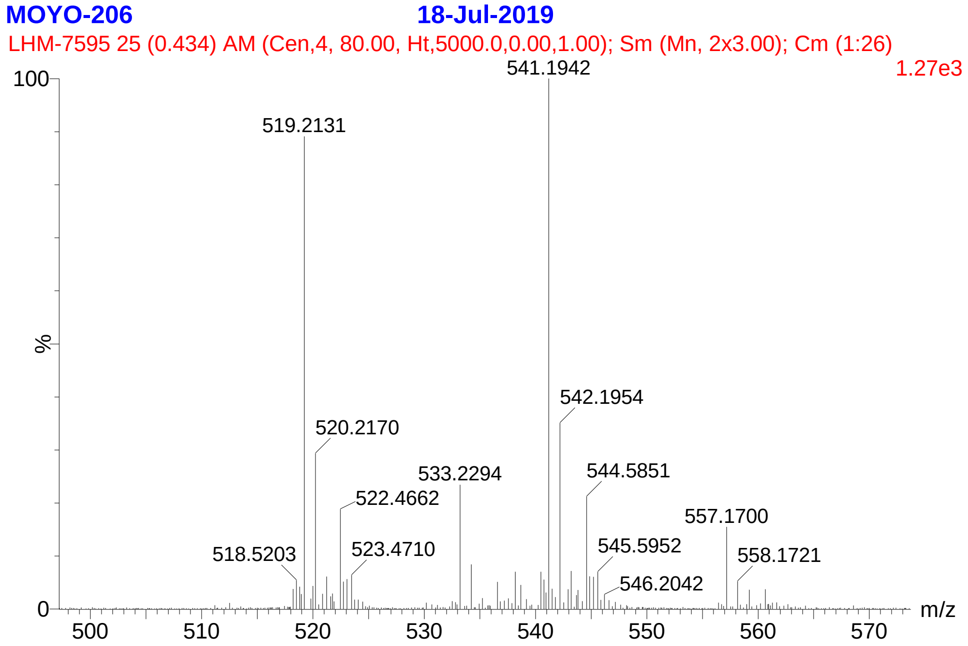

Fig. S14. HRMS spectrum of compound **15e**

^{1}H NMR of compound 15f

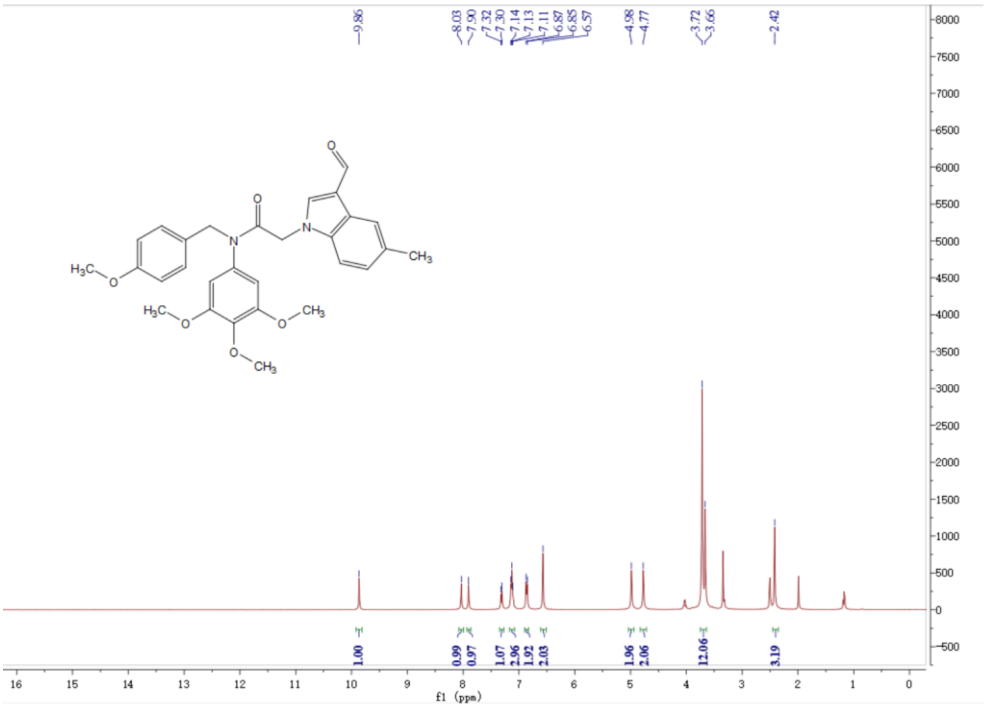

Fig. S15. ^{1}H NMR spectrum of compound **15f** (400 MHz, DMSO-*d6*)

^{13}C NMR of compound 15f

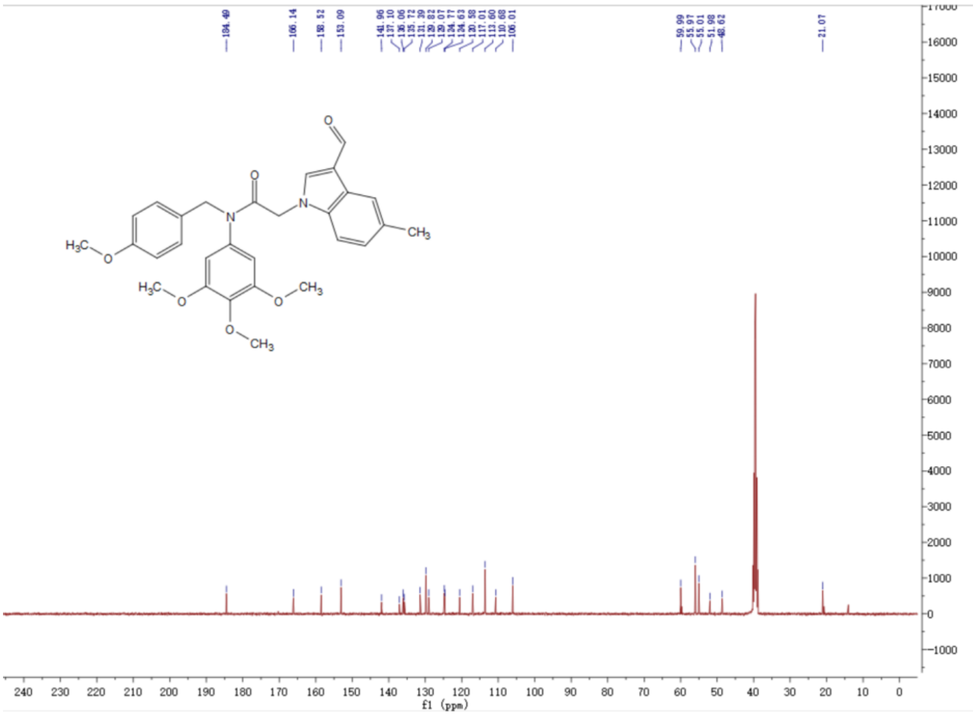

Fig. S16. ^{13}C NMR spectrum of compound **15f** (400 MHz, DMSO-*d6*)

HRMS of compound 15f

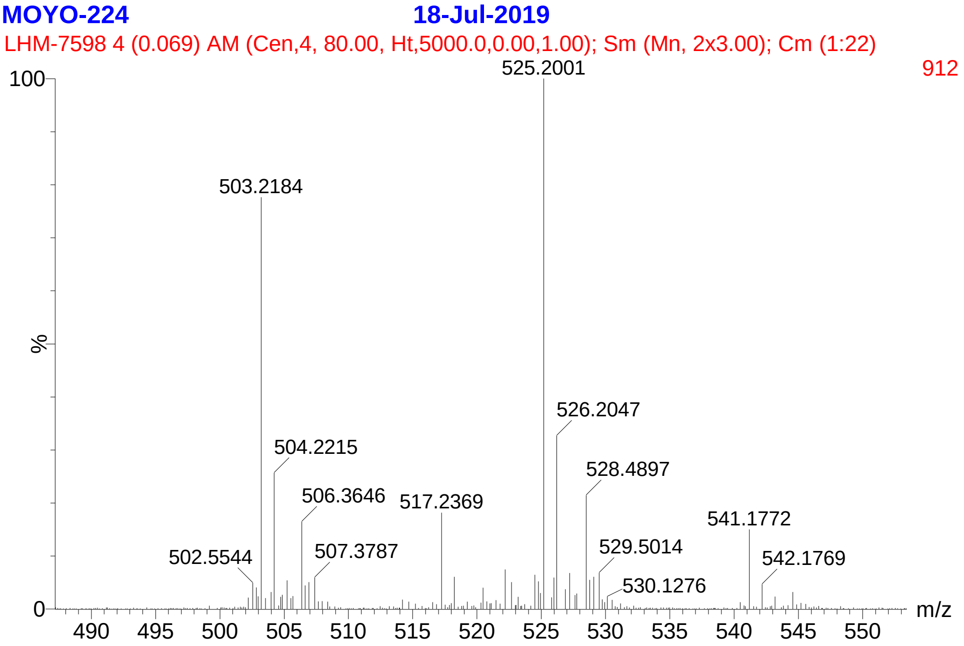

Fig. S17. HRMS spectrum of compound **15f**

^{1}H NMR of compound 15g

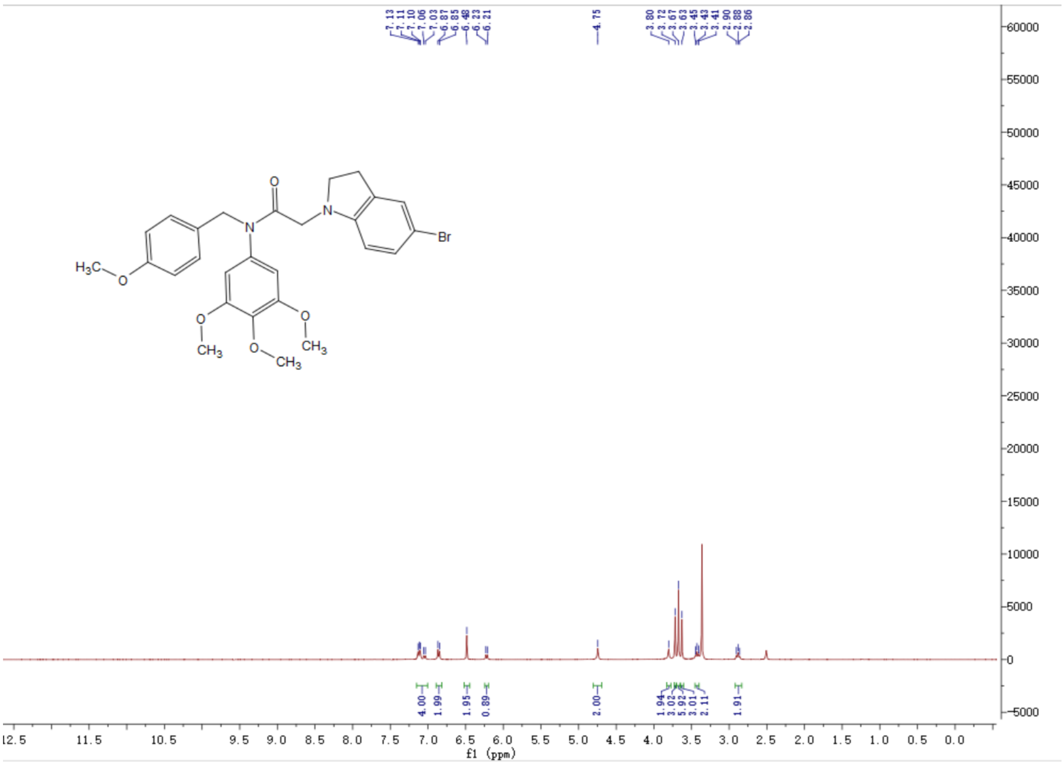

Fig. S18. ^{1}H NMR spectrum of compound **15g** (400 MHz, DMSO-*d6*)

^{13}C NMR of compound 15g

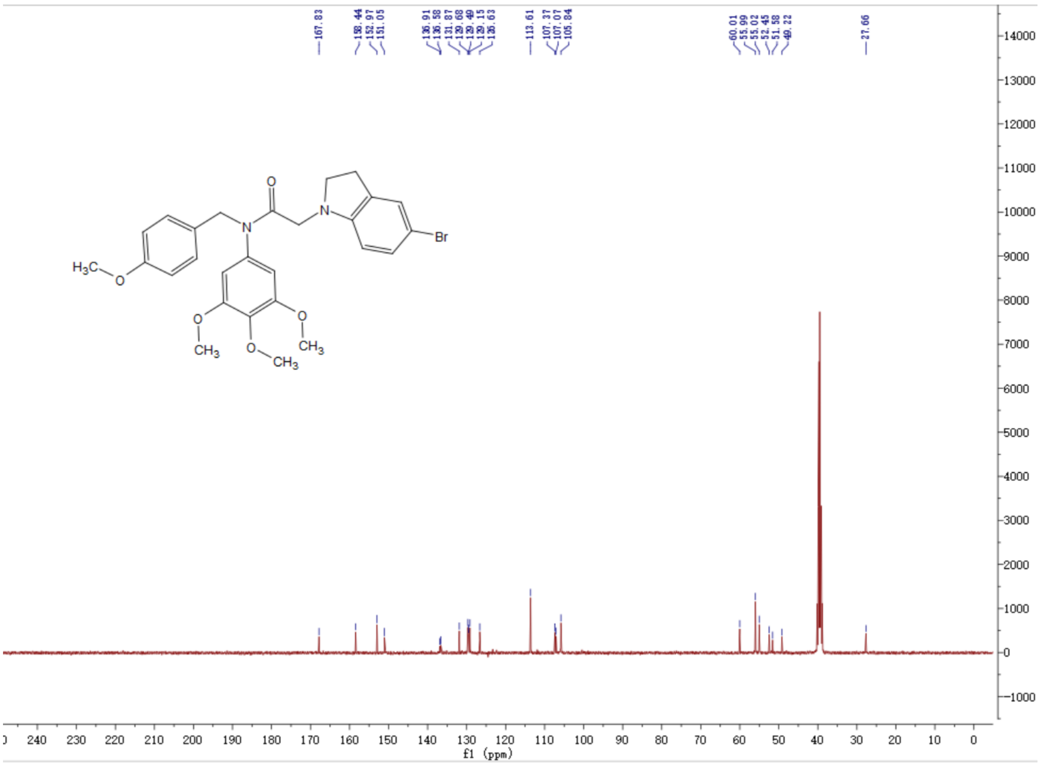

Fig. S19. ^{13}C NMR spectrum of compound **15g** (400 MHz, DMSO-*d6*)

HRMS of compound 15g

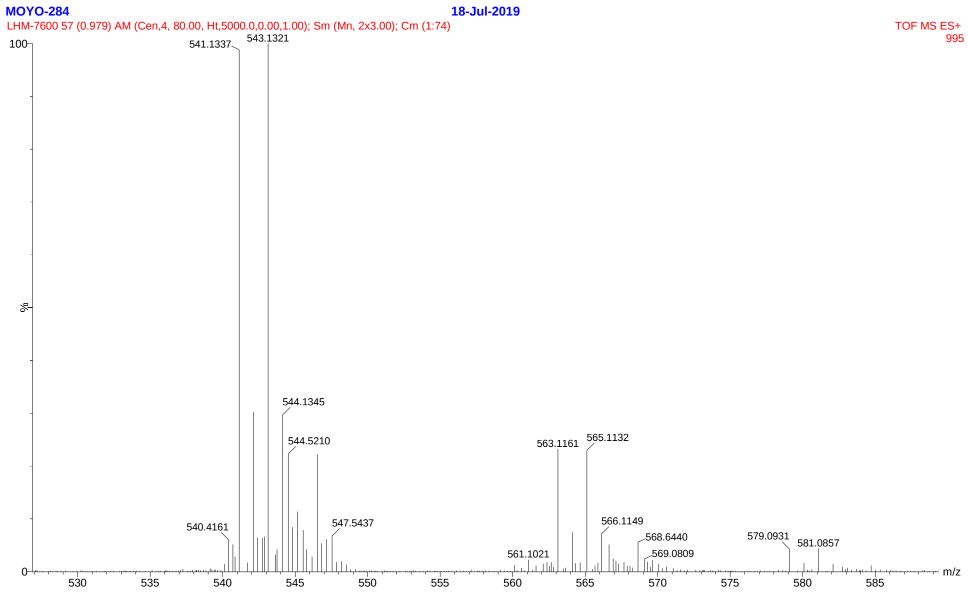

Fig. S20. HRMS spectrum of compound **15g**

^{1}H NMR of compound 15h

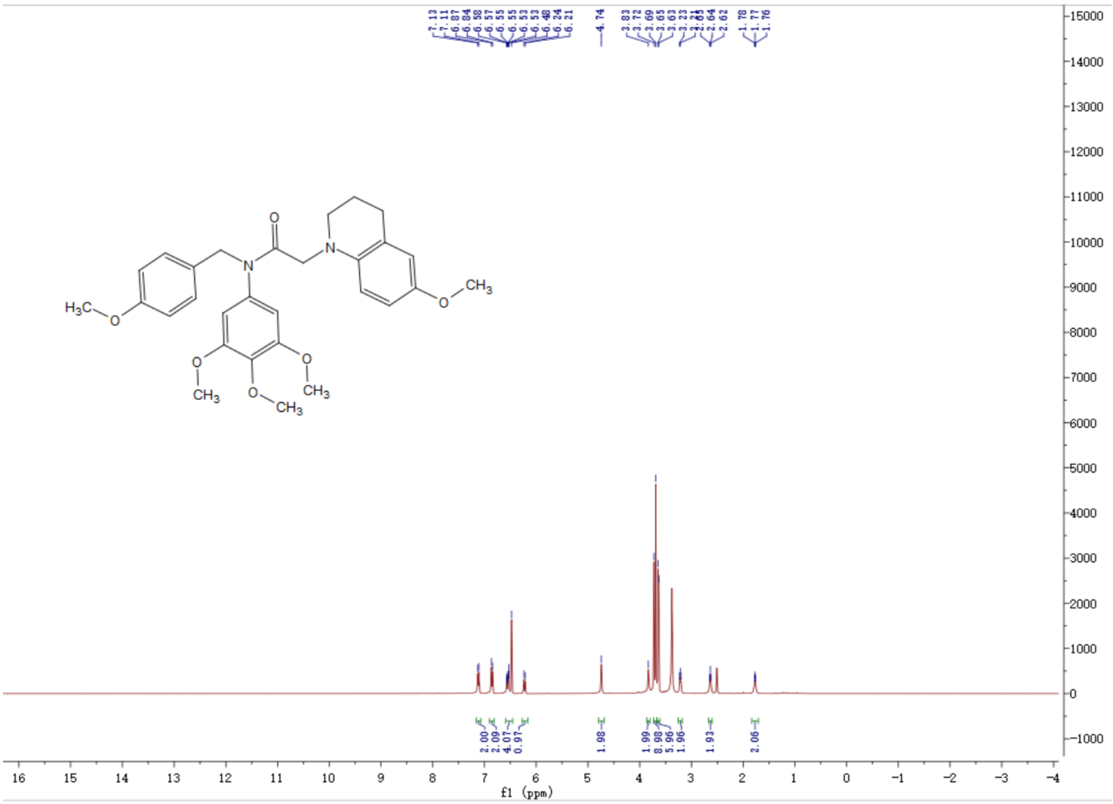

Fig. S21. ^{1}H NMR spectrum of compound **15h** (400 MHz, DMSO-*d6*)

^{13}C NMR of compound 15h

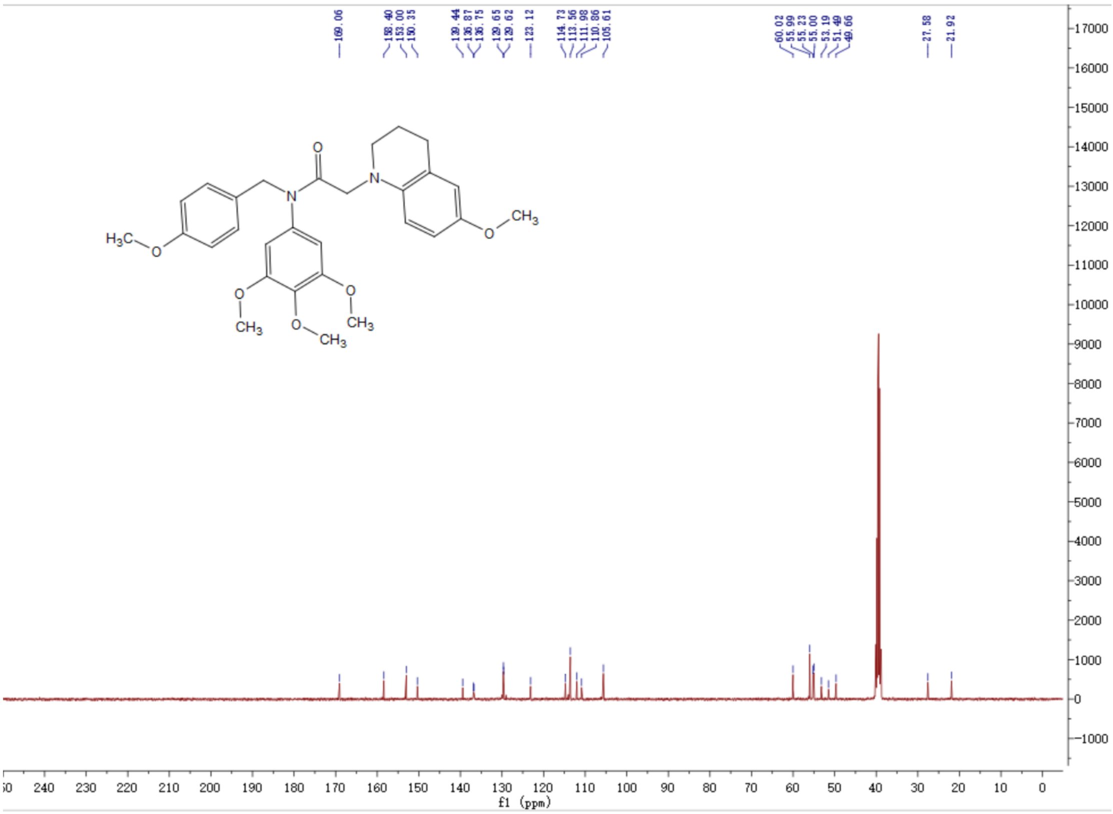

Fig. S22. ^{13}C NMR spectrum of compound **15h** (400 MHz, DMSO-*d6*)

HRMS of compound 15h

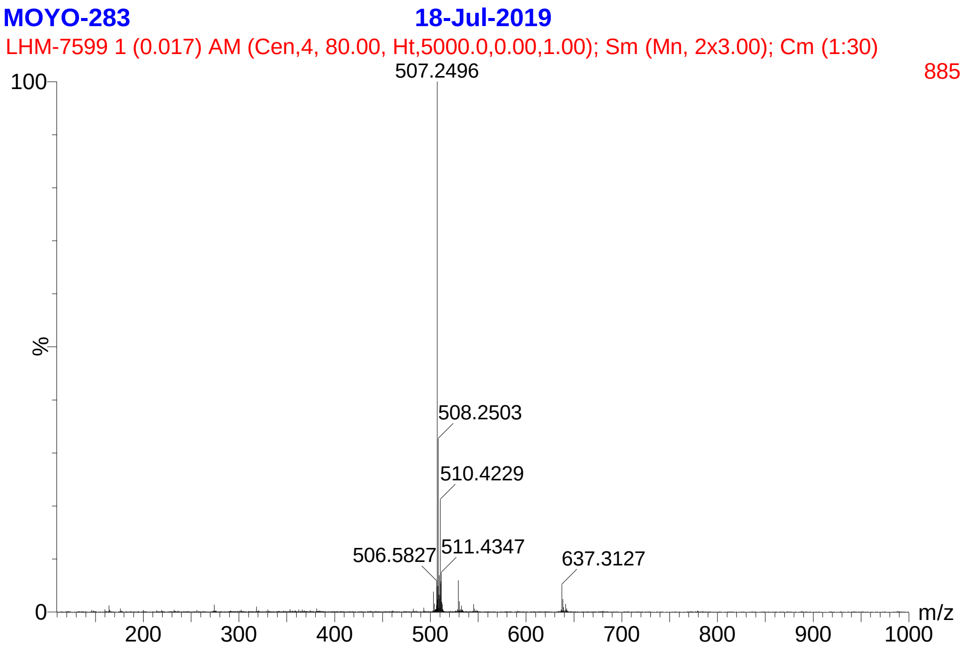

Fig. S23. HRMS spectrum of compound **15g**

^{1}H NMR of compound 19a

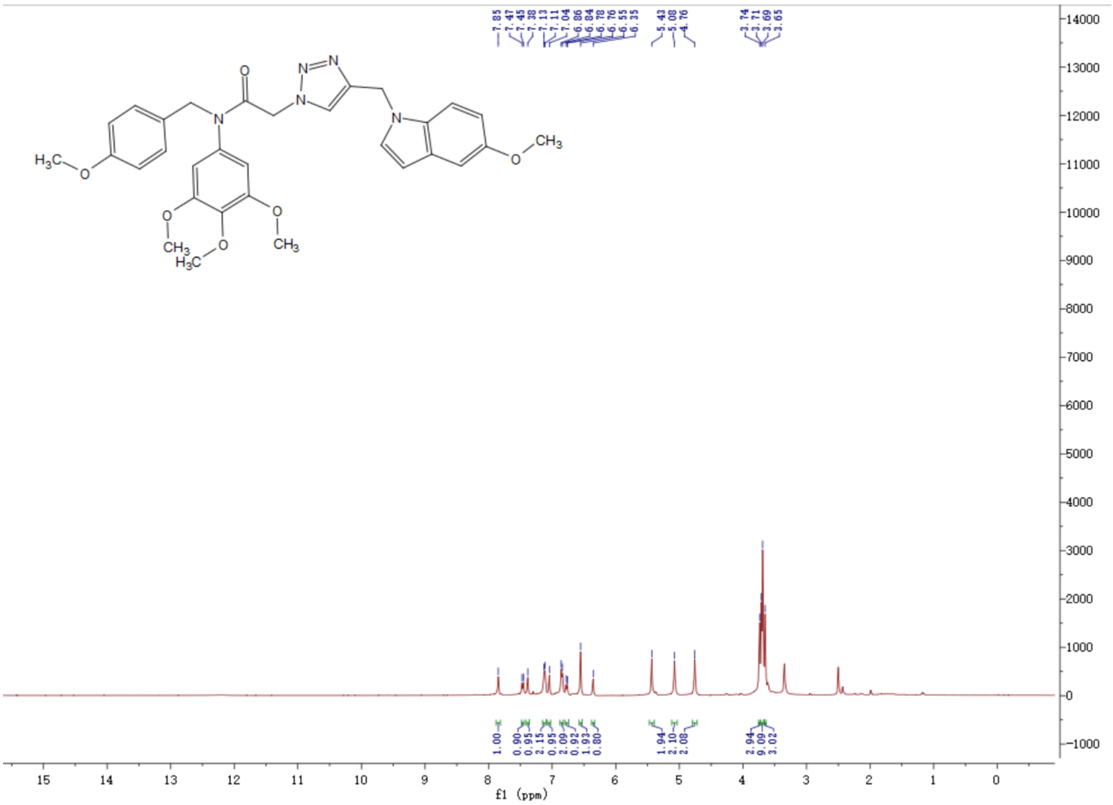

Fig. S24. ^{1}H NMR spectrum of compound **19a** (400 MHz, DMSO-*d6*)

^{13}C NMR of compound 19a

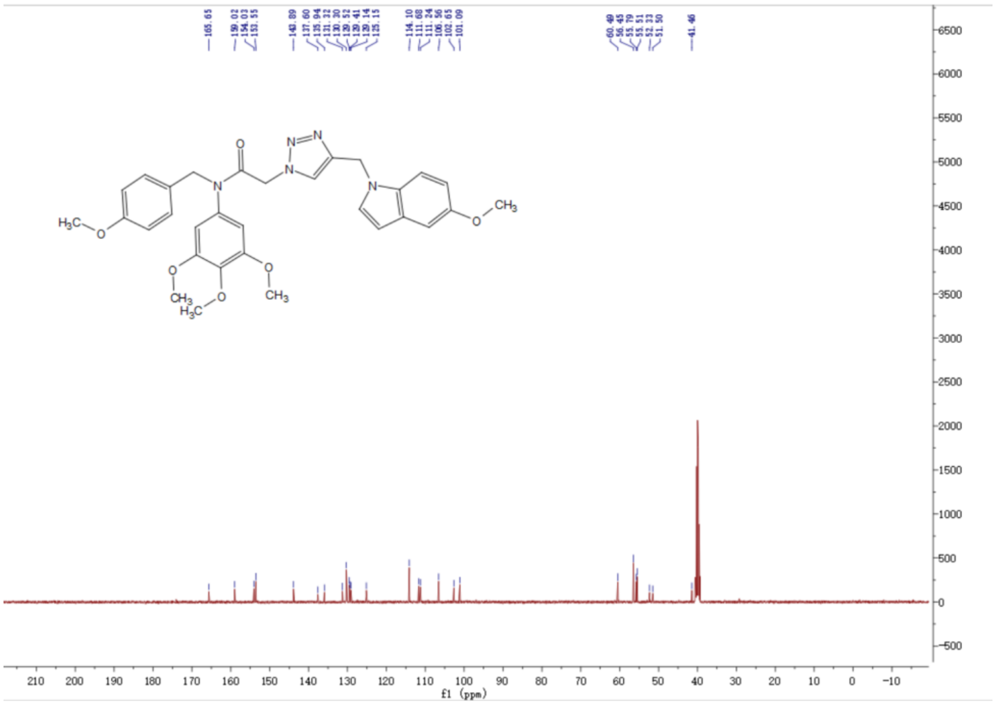

Fig. S25. ^{13}C NMR spectrum of compound **19a** (400 MHz, DMSO-*d6*)

HRMS of compound 19a

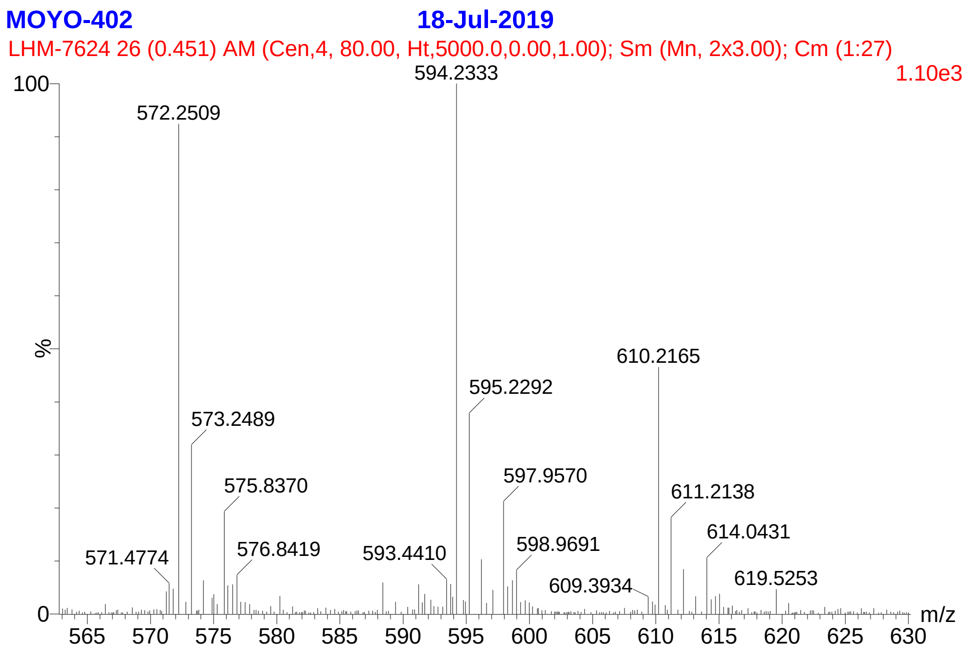

Fig. S26. HRMS spectrum of compound **19a**

^{1}H NMR of compound 19b

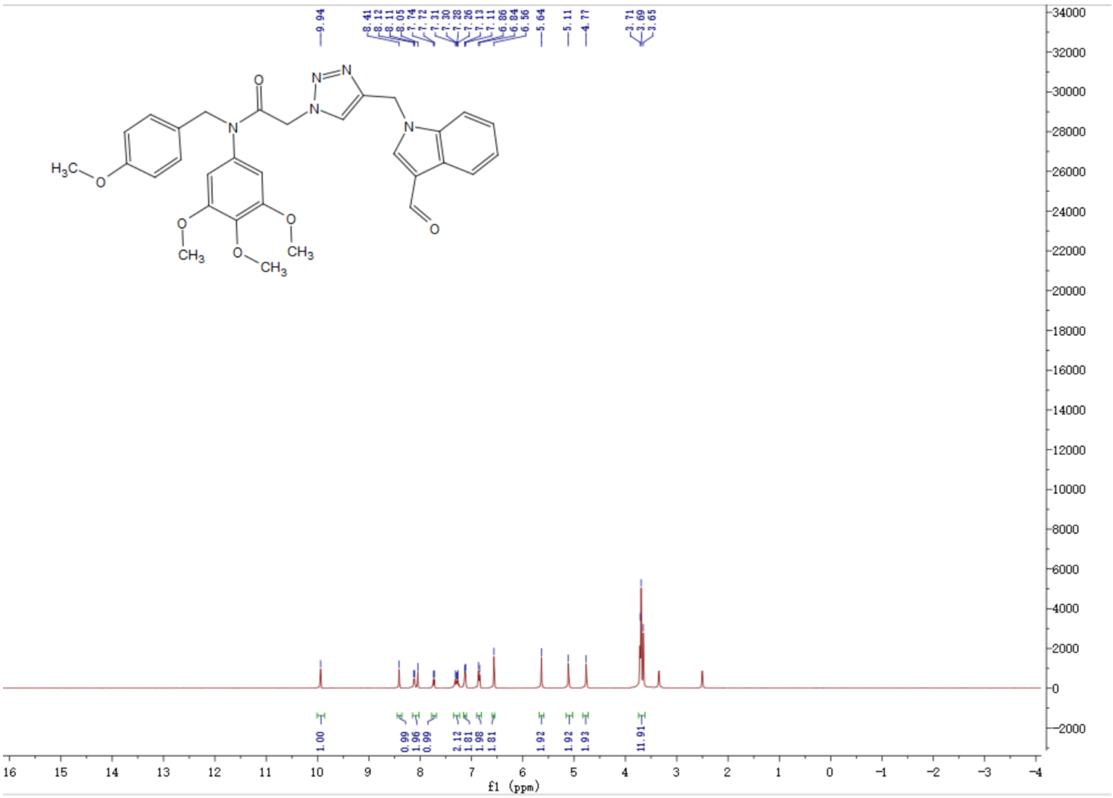

Fig. S27. ^{1}H NMR spectrum of compound **19b** (400 MHz, DMSO-*d6*)

13C NMR of compound 19b

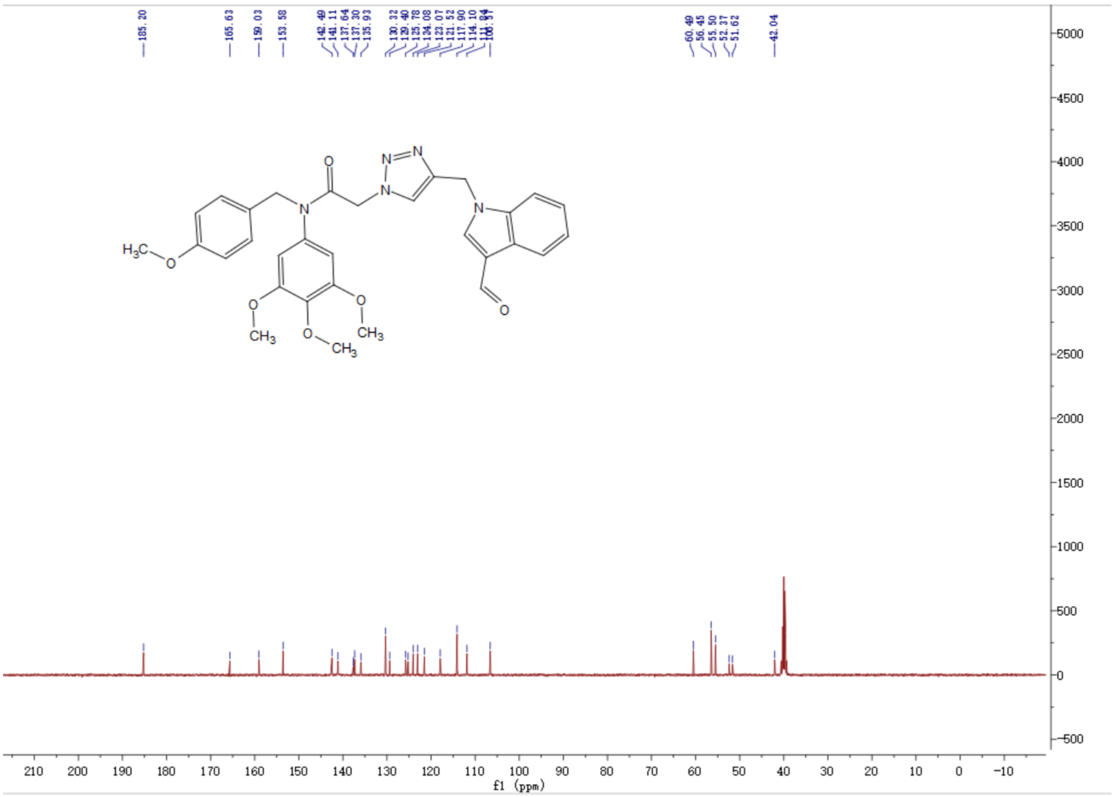

Fig. S28. ^{13}C NMR spectrum of compound **19b** (400 MHz, DMSO-*d6*)

HRMS of compound 19b

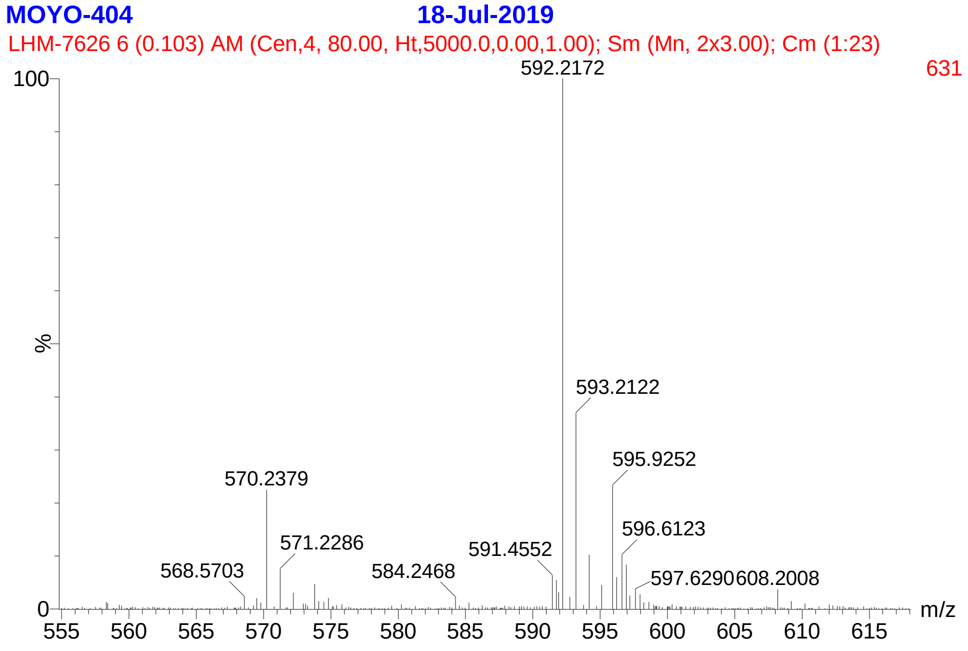

Fig. S29. HRMS spectrum of compound **19b**

^{1}H NMR of compound 19c

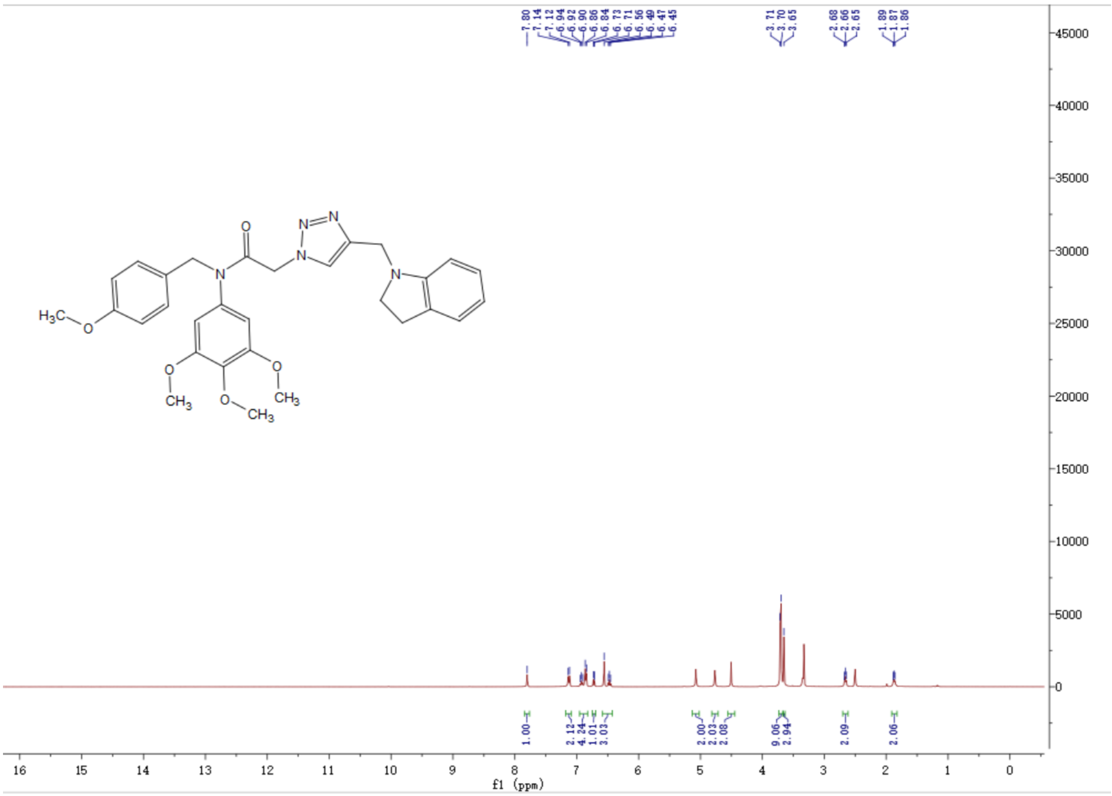

Fig. S30. ^{13}C NMR spectrum of compound **19c** (400 MHz, DMSO-d6)

^{13}C NMR of compound 19c

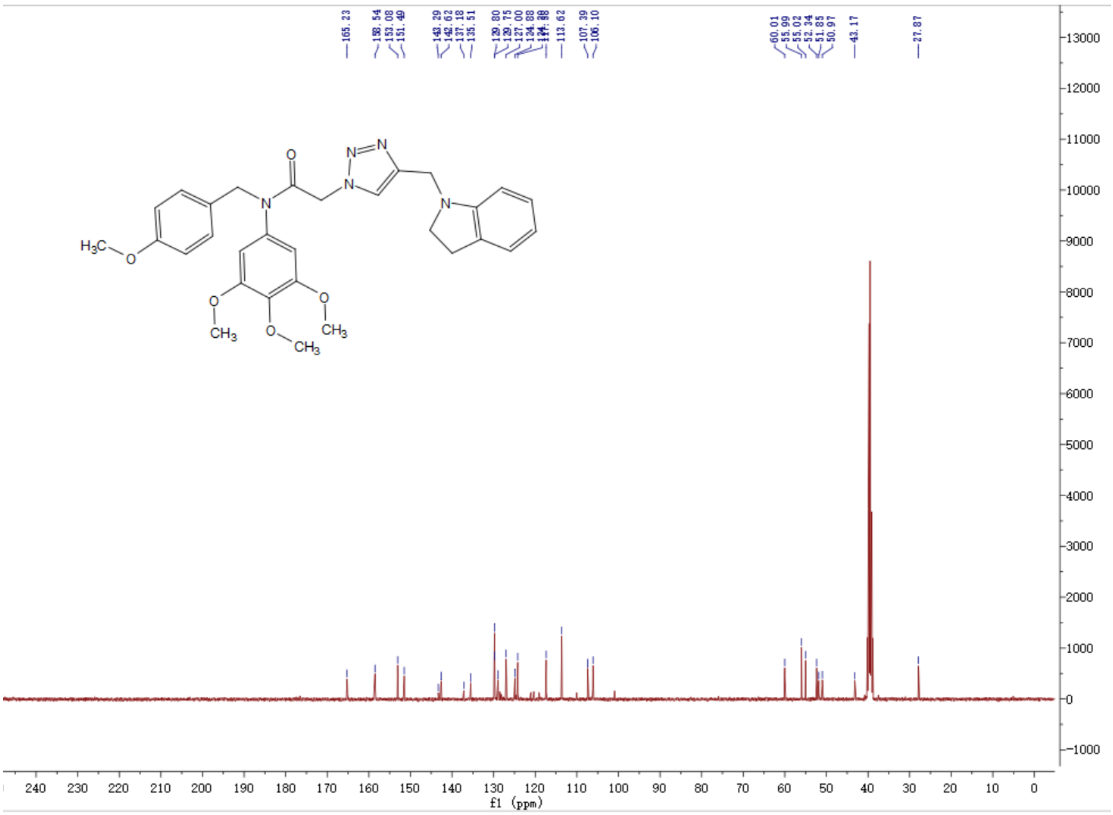

Fig. S31. ^{13}C NMR spectrum of compound **19c** (400 MHz, DMSO-*d6*)

HRMS of compound 19c

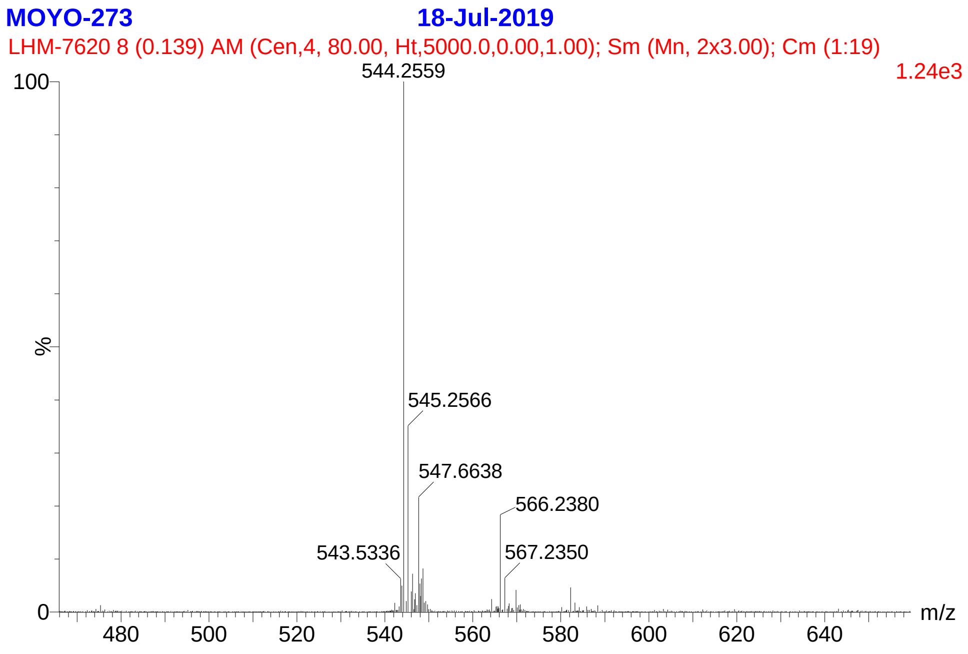

Fig. S32. HRMS spectrum of compound **19c**

^{1}H NMR of compound 19d

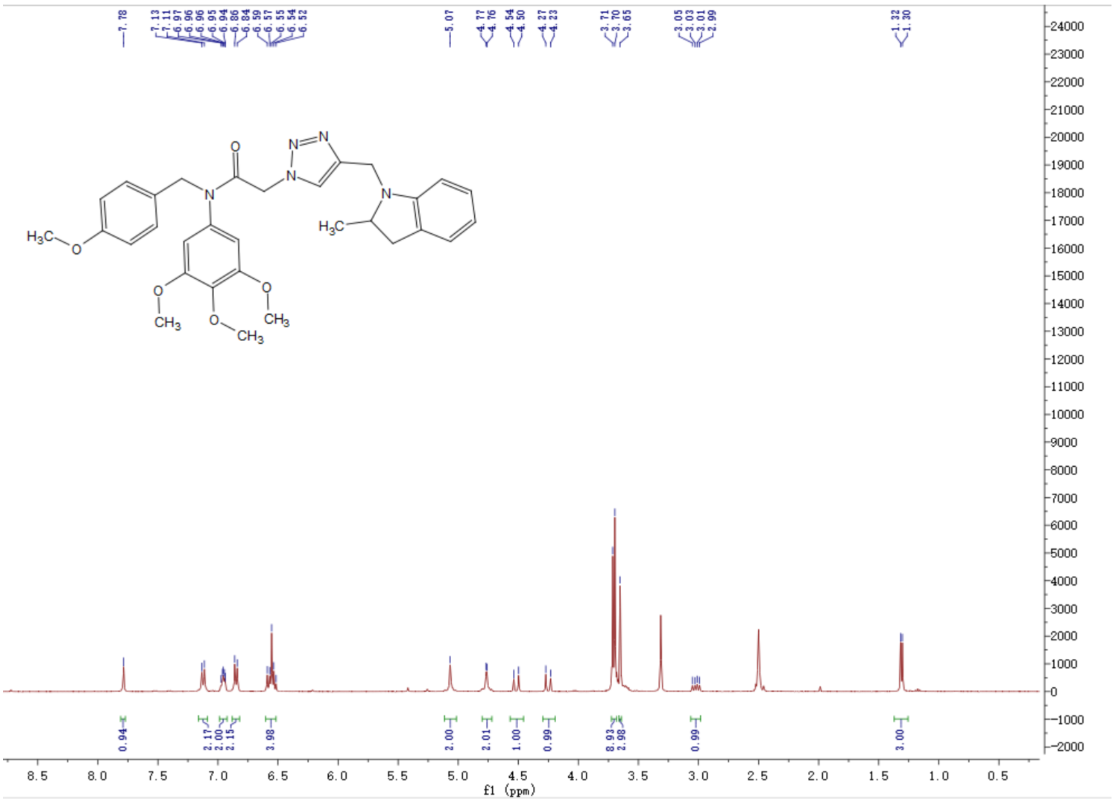

Fig. S33. ^{1}H NMR spectrum of compound **19d** (400 MHz, DMSO-*d6*)

^{13}C NMR of compound 19d

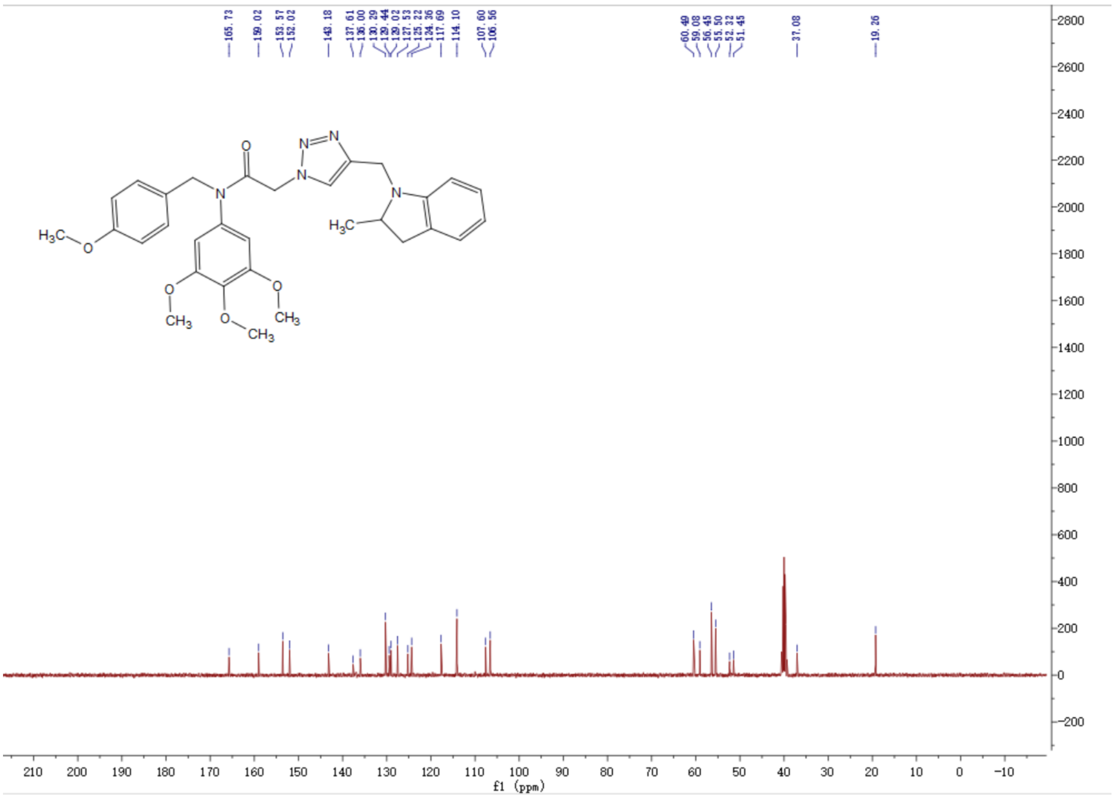

Fig. S34. ^{13}C NMR spectrum of compound **19d** (400 MHz, DMSO-*d6*)

HRMS of compound 19d

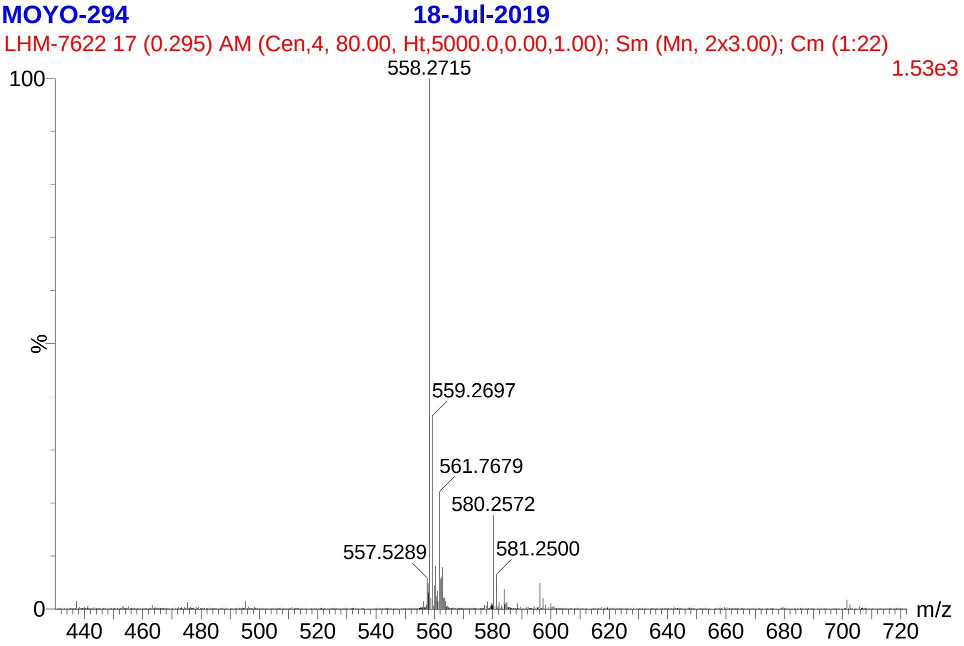

Fig. S35. HRMS spectrum of compound **19d**

^{1}H NMR of compound 19e

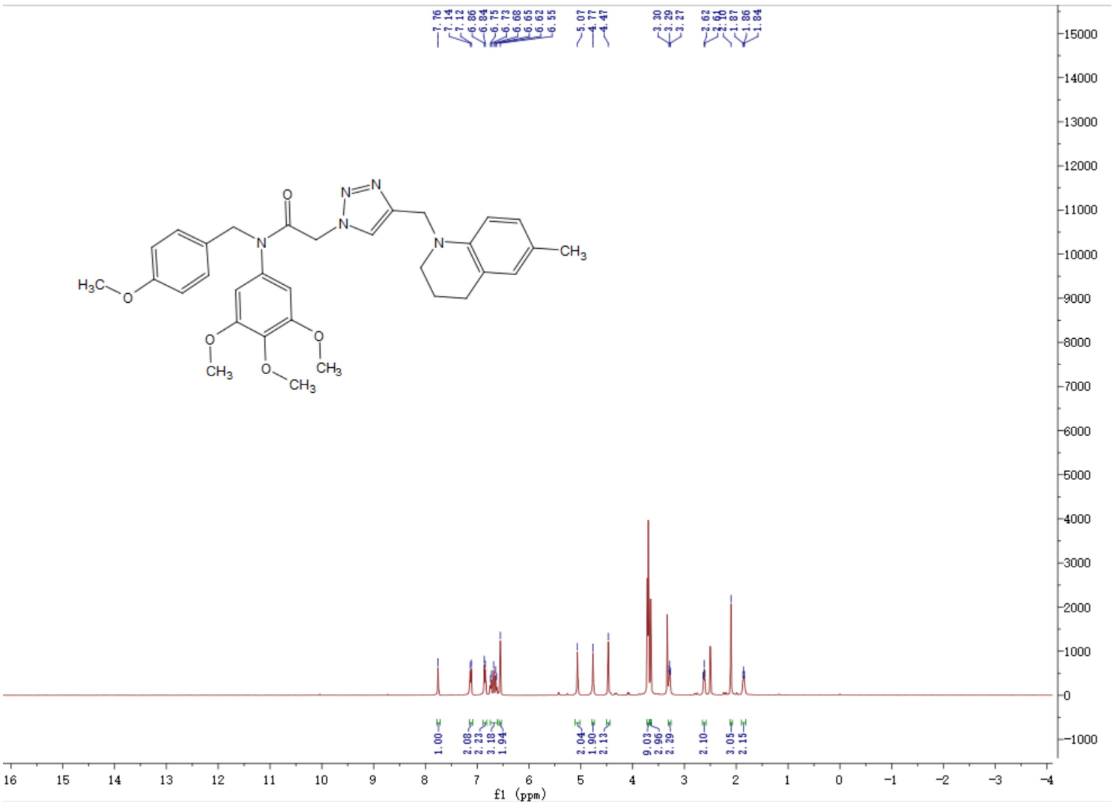

Fig. S36. ^{1}H NMR spectrum of compound **19e** (400 MHz, DMSO-*d6*)

^{13}C NMR of compound 19e

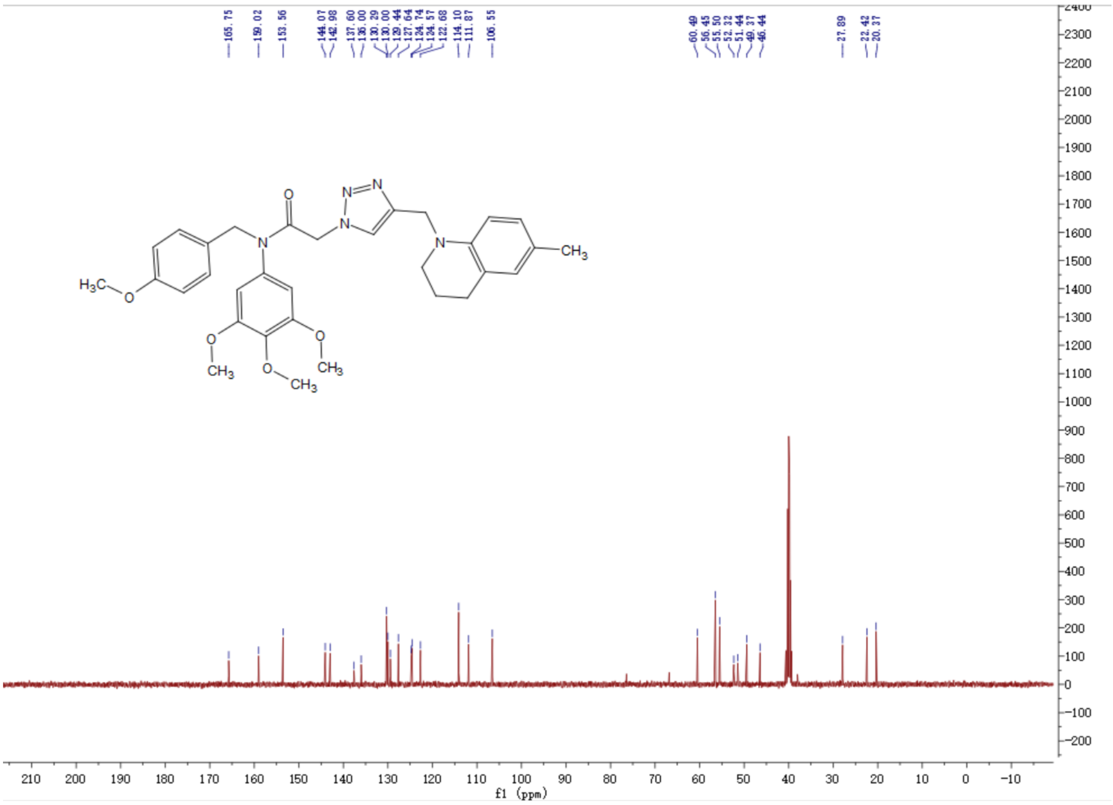

Fig. S37. ^{13}C NMR spectrum of compound **19e** (400 MHz, DMSO-*d6*)

HRMS of compound 19e

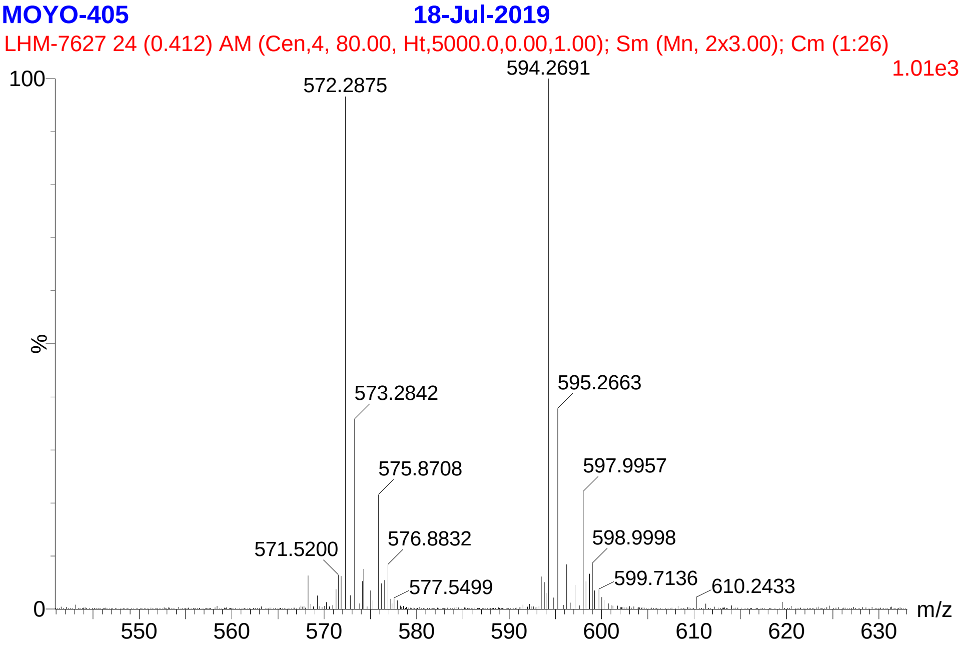

Fig. S38. HRMS spectrum of compound **19g**
